# Estimating conflict losses and reporting biases

**DOI:** 10.1073/pnas.2307372120

**Published:** 2023-08-14

**Authors:** Benjamin J. Radford, Yaoyao Dai, Niklas Stoehr, Aaron Schein, Mya Fernandez, Hanif Sajid

**Affiliations:** ^a^Public Policy Program, University of North Carolina at Charlotte, Charlotte, NC 28223; ^b^Intelligence Community Center of Academic Excellence, Department of Political Science & Public Administration, University of North Carolina at Charlotte, Charlotte, NC 28223; ^c^Department of Political Science & Public Administration, University of North Carolina at Charlotte, Charlotte, NC 28223; ^d^Department of Computer Science, ETH Zürich, Zürich 8092, Switzerland; ^e^Department of Statistics, University of Chicago, Chicago, IL 60637

**Keywords:** news bias, war, casualties, open-source data, Bayesian statistics

## Abstract

Determining the number of casualties and fatalities suffered in militarized conflicts is important for conflict measurement, forecasting, and accountability. However, given the nature of conflict, reliable statistics on casualties are rare. Countries or political actors involved in conflicts have incentives to hide or manipulate these numbers, while third parties might not have access to reliable information. For example, in the ongoing militarized conflict between Russia and Ukraine, estimates of the magnitude of losses vary wildly, sometimes across orders of magnitude. In this paper, we offer an approach for measuring casualties and fatalities given multiple reporting sources and, at the same time, accounting for the biases of those sources. We construct a dataset of 4,609 reports of military and civilian losses by both sides. We then develop a statistical model to better estimate losses for both sides given these reports. Our model accounts for different kinds of reporting biases, structural correlations between loss types, and integrates loss reports at different temporal scales. Our daily and cumulative estimates provide evidence that Russia has lost more personnel than has Ukraine and also likely suffers from a higher fatality to casualty ratio. We find that both sides likely overestimate the personnel losses suffered by their opponent and that Russian sources underestimate their own losses of personnel.

In February 2022, Russian armed forces invaded Ukraine, expanding upon their previous annexation of Crimea and eastern parts of the country in 2014. Since that time, governments, NGOs, and open-source investigators (OSI) have produced thousands of estimates related to physical losses suffered by belligerents in the conflict: casualties, fatalities, and equipment losses. These reported losses form an incomplete time series that provides snapshots of the conflict up until the point at which the claim is made. However, these reported losses are also obscured by the fog of war and often contradict one another. Contemporaneous reported numbers of cumulative incurred losses made by different sources may differ by orders of magnitude. For example, on September 21, 2022, Russian Defense Minister Shoigu reported that 5,937 Russian soldiers had been killed in the conflict (1). However, during the same week, the Ukrainian Ministry of Defense reported that 55,510 Russian soldiers had been killed (2).

We construct a dataset of reports of losses suffered by Russia and Ukraine to predict the daily losses per side and per loss category, where categories include various types of equipment as well as personnel. Furthermore, we account for correlations between loss categories to adjust for gaps in reporting while also accounting for source-specific biases in the original reporting. Under this model, we can predict the expected losses suffered by both sides of the conflict for every loss category at the daily and cumulative levels. We find evidence that Russia has lost substantially more personnel than has Ukraine and also likely suffers from a higher fatality to casualty ratio. However, relative equipment losses tend to be closer to parity between sides. We also find that Russian sources overestimate Ukrainian personnel losses while underestimating their own.

Measuring the casualty and fatality rates of a military conflict is important both for characterizing the conflict and for forecasting its progression. Many definitions of war, for instance, depend on knowledge of both the absolute and relative number of combatant fatalities among belligerents (3). Fatalities themselves are sometimes used as a (near-)continuously valued proxy for concepts that are difficult to measure, like conflict severity (4) or conflict escalation (5).

Assessments of fatality and casualty rates and the number and types of equipment available to the opposing side are crucial for war planning, managing public opinion, and the protection of human rights. There is a long history of statistical modeling in the service of estimating the costs of war. In World War 2, statisticians used consecutive serial numbers on captured German tanks to estimate monthly tank production capacity (“the German tank problem”) with greater accuracy than intelligence analysts (6). At home, political leaders have an interest not only in understanding losses but in managing the public’s understanding of losses. In the context of the United States, the public conditioned its support for military action against Iraq, at least in part, on perceptions of the expected casualty numbers (7). The importance of public perceptions of losses during a military conflict is underscored by media reports from April 2023: Leaked US intelligence documents apparently revealed internal estimates of Ukrainian and Russian losses during the ongoing conflict. However, reports also indicate that the numbers in the documents were likely modified by a third party at some point to minimize Russia’s losses (8). Recent work also suggests that governments will underreport violence against noncombatants (9). This highlights the importance of accounting for the biases of specific sources when estimating losses during military conflicts.

Unfortunately, as Clausewitz writes, “casualty reports on either side are never accurate, seldom truthful, and in most cases deliberately falsified” (10). Accurate casualty estimates are “notoriously difficult” (11). Methods for estimating war deaths can be grouped into two primary categories: Those that rely on contemporaneous reports, often from multiple sources, and those that rely on postwar surveys or demographic studies (12). One notable effort to catalog combat-related deaths is the PRIO Battledeaths Dataset (13). This effort, like ours, falls primarily within the former estimation methodology. Unlike our approach, the PRIO Battledeaths Dataset reports only annual battle-related deaths per conflict per country. In contrast, we attempt to leverage the temporal aspect of casualty reports to estimate losses at the daily level. We also leverage reports of multiple loss categories to help fill gaps in reporting for any single loss category, under the assumption that losses in some categories will be correlated with others. For example, losses of armored vehicles are likely correlated with casualties (14).

Our data contain 4,609 claims of losses reported on social media, news venues, and by various government sources. We aggregate these sources into seven groups that we refer to as “claim sources”: OSI (n=169), Russian sources (247), UK sources (32), Ukrainian sources (3,858), the United Nations (78), US sources (71), and other sources (154).^*^ Losses are recorded at two levels, daily and cumulative, with the latter comprising 96.5% of all observations. We record losses for 21 categories, 14 of which are given in Table 1 and include military and civilian fatalities, casualties, and losses of various types of equipment. Due to reporting inconsistencies, all time series are incomplete and many contain inconsistent observations: nonmonotonically increasing cumulative values, missing values, and multiple contradictory values on a single day. We use all available data to estimate daily and cumulative losses for all loss categories while accounting for claim source–specific biases in the reports.

**Table 1.** Estimated cumulative losses as of February 23, 2023

ISO2	Category	n	Est.	95% CI
RU	AA Systems	233	339	[76–1,070]
UA	AA Systems	13	1,105	[108–5,247]
RU	Armored Vehicles	400	6,351	[2,966–11,791]
UA	Armored Vehicles	15	3,280	[777–8,439]
RU	Artillery	380	1,483	[701–2,818]
UA	Artillery	35	2,290	[519–6,966]
UA	Civilian Casualties	21	38,155	[13,245–84,852]
UA	Civilian Deaths	46	13,287	[4,081–32,399]
UA	Civilian Injuries	26	19,464	[5,396–46,460]
RU	Helicopters	389	172	[87–311]
UA	Helicopters	30	64	[14–183]
RU	Jets	409	146	[68–273]
UA	Jets	38	122	[32–372]
RU	Military Casualties	130	218,800	[108,432–397,361]
UA	Military Casualties	16	75,538	[19,994–176,612]
RU	Military Deaths	523	76,687	[38,670–139,772]
UA	Military Deaths	67	17,223	[6,219–39,105]
RU	Military Injuries	44	148,608	[45,749–365,649]
UA	Military Injuries	8	33,081	[5,260–125,925]
RU	MLRS	261	488	[148–1,222]
UA	MLRS	27	538	[155–1,482]
RU	Tanks	501	3,380	[1,704–6,178]
UA	Tanks	33	2,051	[385–5,946]
RU	UAVs	292	337	[153–707]
UA	UAVs	40	1,643	[387–4,371]

Note that for some loss categories, Feb. 23, 2023, may be many months beyond the latest observed report. Loss types with few data or that represent composite categories are omitted for concision.

## Results

**Fig. 1.**
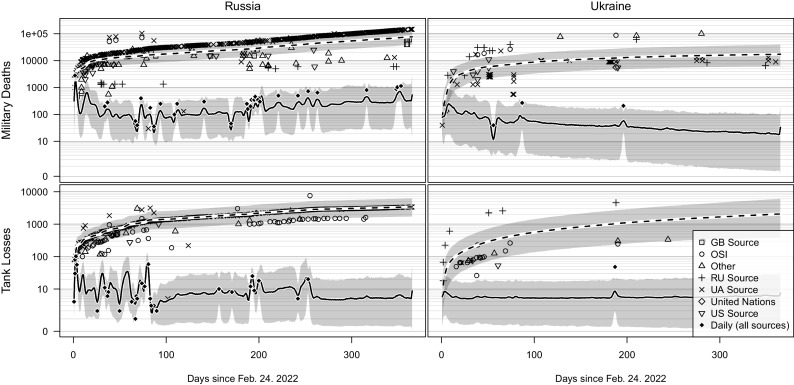
Estimated military personnel (*Top*) and tank (*Bottom*) losses incurred by Russia (*Left*) and Ukraine (*Right*) during the first 365 d of war. Expected daily losses are depicted with a solid line, and expected cumulative losses are depicted with a dashed line. Note the 95% posterior credible intervals in gray. Points indicate reports from the sources given in the legend in the *Lower right*. *Y*-axis values are $\sinh^{-1}\left(y\right)$ transformed for clarity.

With our model, we estimate expected daily and cumulative losses for every loss category and target country pair (“category–target”), conditioned on estimated claim source biases. Fig. 1 depicts the data and posterior predictions for military personnel deaths and tank losses for both major parties to the conflict. We find that Russian personnel losses have outpaced Ukrainian personnel losses, with expected loss numbers of 76,687 (95% credible interval: 38,670–139,772) and 17,223 (6,219–39,105), respectively, as of February 23, 2023. We compute the ratio of casualties to deaths for Russia and Ukraine, finding values of 2.9:1 and 4.9:1, respectively.

In the lower two panels of Fig. 1, we see expected losses of tanks over time. Both Russia and Ukraine are estimated to have suffered similarly here, with expected losses of 3,380 and 2,051, respectively. The uncertainty, indicated visually by the gray-shaded 95% posterior credible intervals, is much higher for Ukraine, though, obtaining lower and upper bounds of 385 and 5,946 by the end of the first year of war, versus Russia’s bounds of 1,704 and 6,178.

Table 1 presents a selection of estimated cumulative losses as of February 23, 2023, alongside the number of reports corresponding to each loss type (n), and the bounds of a 95% posterior credible interval for each estimate.

We also estimate the biases exhibited by claim sources with respect to loss categories. Bias does not necessarily imply intentional misrepresentation but rather any systematic over- or underestimation relative to our estimated loss values. When looking at Russian military deaths, we find that, for every loss suffered, Russian sources report only 0.3 losses (0.1–0.5). This roughly corresponds to the Russian account of 5,937 losses by September 21, 2022, at which point our model estimates Russia had likely lost 31,532 soldiers. Russian sources overestimate Ukrainian military deaths at a rate of 4.3 to 1. Ukrainian sources overestimate Russian deaths by nearly double, though no bias is supported in the 95% CI (1.0:1–3.4:1). We find no evidence of systematic bias in Ukrainian reports of Ukrainian military deaths.

## Discussion

Overall, we find that Russian and Ukrainian equipment losses are often comparable by category, but that Russian personnel losses outpace Ukrainian personnel losses. This may reflect accounts of poorly equipped Russian soldiers and ineffective supply lines leading to relatively higher human costs, a narrative that has been popular in the media. As of the one-year mark, Russia appears to have lost personnel relative to Ukraine at a rate of 5.53 to 1 (1.6:1–14.5:1).

More generally, we have proposed a method for measuring conflict-related losses with high temporal fidelity from open-source data. Our approach deals with source-specific biases in a principled way, treating them as parameters to be estimated. It also incorporates both daily and cumulative reports about multiple distinct loss categories, given as either ranges or point estimates. This allows researchers to leverage the breadth of available reporting when reporting on any single type of loss is likely to be scarce.

## Materials and Methods

We use a single multivariate Bayesian model with two outcomes: daily and cumulative loss counts. Every observation consists of a loss report (either daily or cumulative), the loss report’s “source,” the country to which the loss refers (the “target,” either Russia or Ukraine), the category of the loss (e.g., tanks, helicopters), the day of the reported loss, and whether the report is a range (e.g., “50–100”), lower bound (e.g., “at least 50”), upper bound, or a point estimate. We assume that the outcomes are either Poisson- or negative binomial–distributed with means that are log-linear in covariates. For every category–target pair (e.g., “Tanks-Ukraine”), we also estimate a latent time series of expected daily losses using cubic basis splines.

We assume Poisson and negative binomial distributions for daily and cumulative losses, $y_{i}^{\text{daily}}$ and $y_{j}^{\text{cum}}$, respectively, where i and j index daily and cumulative observations (Eqs. **1** & **2**). The log daily and cumulative mean estimates are shown in Eqs. **3** and **4**. Coefficients are denoted with β. Our estimates of the latent time series for every loss category for every target country are represented by $\theta_{ct,d}$, where ct indexes the category–target and d the number of days since February 24, 2022. The mean daily losses for a given category–target, $\beta_{\mathrm{ct}}^{\text{const}}$, are drawn from a normal distribution (Eq. **6**). This mean is added to a time trend, βcttrendd365, and to time-varying deviations, $B\beta_{\mathrm{ct}}^{\;\text{spline}}$, to capture the temporal dynamics of losses, where $\beta_{\mathrm{ct}}^{\;\text{spline}}$ are spline coefficients and B is a cubic basis spline with 150 kn. Spline coefficients are multivariate normal distributed (Eq. **7**). Every claim source (indexed by s) is given two scalar coefficients to account for minimum and maximum estimates when ranges are given (Eq. **8**). $I_{i}^{\;\text{min}}$ and $I_{i}^{\;\text{max}}$ indicate that observation i is a minimum or maximum estimate. Every observation is scaled by a multilevel bias coefficient that is specific to its source–target pair (indexed by st) and category, $\beta_{c,st}^{\;\text{bias}}$, to account for systematic over- or underestimation. These are normally distributed around source–target specific means which are themselves normally distributed with prior mean zero (Eq. **9**). We assume zero-centered bias terms, encoding the conservative belief that a source is unbiased absent data to indicate otherwise. A nonzero mean would encode belief in a systematic bias (e.g., systematic measurement error). We model bias terms hierarchically to mitigate class imbalances through partial pooling. When estimating losses, we set the bias terms to zero (i.e., “no bias”). Category–target-specific overdispersion is accounted for by $\phi_{\mathrm{ct}}$ (Eq. **10**). For brevity, we omit hyperpriors.


[1] $$y_{i}^{\text{daily}}\sim \text{Pois}(\exp(\mu_{i}^{\text{daily}})),$$

[2] $$y_{j}^{\text{cum}}\sim \text{NB}(\exp(\mu_{j}^{\text{cum}}),1/\exp(\phi_{ct[j]})),$$

[3] $$\mu_{i}^{\text{daily}}=\theta_{ct[i],d[i]}+\beta_{c[i],st[i]}^{\text{bias}}+\beta_{s[i]}^{\text{min}}I_{i}^{\text{min}}+\beta_{s[i]}^{\text{max}}I_{i}^{\text{max}},$$

[4] $$\mu_{j}^{\text{cum}}=\ln(\Sigma_{k=1}^{d[j]}\exp(\theta_{ct[j],d[k]}))+\beta_{c[j],st[j]}^{\text{bias}}+\beta_{s[j]}^{\text{min}}I_{j}^{\text{min}}+\beta_{s[j]}^{\text{max}}I_{j}^{\text{max}},$$

[5] $$\theta_{ct,d}={\left(B\beta_{ct}^{\text{spline}}\right)}_{d}+\beta_{ct}^{\text{const}}+\beta_{ct}^{\text{trend}}\left(d/365\right),$$

[6] $$\begin{array}{ll} \text{Priors} & \beta_{c}^{\text{const}}\sim N(\mu^{\text{const}},\;\sigma^{\text{const}}) \end{array}$$

[7] $$\begin{array}{ll} \beta_{ct}^{\text{trend}}\sim N(\mu^{\text{trend}},\sigma^{\text{trend}}) & \beta_{ct}^{\text{spline}}\sim N(0,\Sigma^{\text{spline}}), \end{array}$$

[8] $$\begin{array}{ll} \beta_{s}^{\text{min}}\sim N(\mu^{\text{min}},\sigma^{\text{min}}) & \beta_{s}^{\text{max}}\sim N(\mu^{\text{max}},\sigma^{\text{max}}), \end{array}$$

[9] $$\begin{array}{ll} \beta_{c,st}^{\text{bias}}\sim N(\gamma_{st}^{\text{bias}},\sigma_{st}^{\text{bias}}) & \gamma_{st}^{\text{bias}}\sim N(0,\sigma_{1}^{\text{bias}}), \end{array}$$

[10] $$\begin{array}{ll} & \phi_{ct}\sim N(\mu^{\phi },\sigma^{\phi }), \end{array}$$

## Footnotes

^*^Source refers to the originator of a claim (e.g., US Department of Defense or Russian Ministry of Defense) and is distinct from the venue that reported on a claim (e.g., CNN or NYT).
